# Role of Zoledronic Acid Supplementation in Reducing Post-Surgical Recurrence of Giant Cell Tumor of Bone: A Meta-Analysis of Comparative Studies

**DOI:** 10.7759/cureus.16742

**Published:** 2021-07-29

**Authors:** Arvind Kumar, Siddhartha Sinha, Yawar Haider, Javed Jameel, Sandeep Kumar

**Affiliations:** 1 Orthopaedics, Hamdard Institute of Medical Sciences and Research, New Delhi, IND

**Keywords:** adjuvant therapy, giant cell tumor, tumor recurrence, zoledronate, zoledronic acid

## Abstract

Zoledronic acid is a bisphosphonate that has recently gained interest in adjuvant therapy for giant cell tumor of bone (GCTB). It has an apoptotic effect on osteoclasts that are precursors of GCTB. However, the evidence suggesting the role of zoledronic acid in preventing GCTB recurrence is mixed, and therefore, a consensus is yet to be established. The purpose of the current meta-analysis was to analyze the impact of zoledronic acid supplementation on tumor recurrence in surgical treated GCTB.

A systematic search was conducted on PubMed, Embase, and Web of Science databases to identify studies that analyzed the impact of local or systemic zoledronic acid supplementation on clinical outcomes in surgically treated GCTB. The data from the comparative studies were pooled and analyzed to investigate the association of zoledronic acid supplementation with tumor recurrence. Additionally, other factors such as age, gender, soft tissue extension, polymethyl methacrylate (PMMA) cement application, recurrent presentation, and extended curettage were also investigated for any association with tumor recurrence.

Of the 271 results, 13 unique studies reported the clinical outcomes in GCTB. Seven studies compared the outcomes of zoledronic acid supplementation with control groups. Six studies presented the tumor recurrence-related data among the comparison groups. The zoledronic acid supplementation was associated with significantly lower tumor recurrence rates (p = 0.007). Additionally, a significant association of soft tissue extension and non-usage of PMMA cement with tumor recurrence were observed.

The current meta-analysis suggests that zoledronic acid supplementation reduces tumor recurrence rates in surgically treated GCTB. We, therefore, recommend the use of zoledronic acid following aggressive extended curettage of the tumor. Further, well-planned randomized controlled trials will help strengthen this evidence.

## Introduction and background

The giant cell tumor of bone (GCTB) is a locally aggressive tumor of bone that usually affects the epiphysial-metaphyseal region of the bones. Although uncommon, the management of GCTB is often challenging and fraught with complications of extensive osseous destruction, local recurrences, and rarely metastasis [1]. These tumors commonly affect the distal femur and proximal tibia and often present with extensive juxta-articular destruction. For the tumors with preserved subchondral bone, joint salvage is possible with the extended curettage of the tumor cavity. For tumors with extensive subchondral damage or joint penetration, endoprosthesis-based reconstruction is desirable [2,3]. The high recurrence rate in GCTB has been reduced by the advent of extended curettage techniques that use chemical agents like phenol, alcohol, or liquid nitrogen.

Additionally, high-speed burrs for intralesional clearance of loculi and bone and the thermogenic effect of polymethyl methacrylate (PMMA) cement have been utilized [4]. As a result, the tumor recurrence rates, which used to be more than 50%, have now been reduced to the order of 20%-30% with extended curettage techniques. Recently, systemic and local adjuvant therapies have been advocated to reduce the tumor recurrence risk [5]. Bisphosphonates and denosumab are the two main candidates that inhibit osteoclasts at the molecular level. The widely studied bisphosphonate for its effect on neoplastic cells in GCTB, zoledronic acid, has been shown to induce neoplastic stromal cell inhibition, apoptosis, and osteogenic differentiation [6]. Denosumab is a receptor activator of nuclear factor κB-ligand (RANKL) inhibitor and was initially advocated for advanced or inoperable and metastatic GCTB [7]. Denosumab is a fully human monoclonal RANKL antibody that targets and binds with high affinity to RANKL, preventing its binding to the RANK receptor on the surface of osteoclast precursors and osteoclasts, thereby inhibiting osteoclast differentiation, activation, and survival. Although the early results with denosumab were encouraging as the surgery became easier due to ossification of the tumor lining, concerns have been raised for higher risk of tumor recurrence as it becomes difficult to distinguish tumor borders from healthy bone and trapped residual neoplastic cells in the new bone [8-11].

The currently reported literature concerning the effectiveness of zoledronic acid in reducing recurrence is heterogenous considering the varying level of evidence and the outcomes. Therefore, we present a meta-analysis of the comparative studies that analyzed the impact of zoledronic acid supplementation in surgical treated GCTB in terms of tumor recurrence.

## Review

We performed a meta-analysis to address the aforestated purpose according to the Cochrane Handbook Oof Systematic Review and Meta-Analysis of Interventions.

Searching strategy

Following the preferred reporting items for systematic reviews and meta-analyses (PRISMA) guidelines, two authors independently searched the PubMed, Embase, and Web of Science databases on April 5, 2021, using the following keywords: giant cell tumor, giant cell tumour, GCT, osteoclastoma, zoledronic acid, and zoledronate. Additionally, a manual search was performed by scrutinizing bibliographies of publications identified for additional articles. Finally, the Cochrane Central Register of Controlled Trials (CENTRAL) was searched to identify any unpublished or ongoing trials. The search strategy was not restricted to the year of publication or language.

Inclusion and Exclusion Criteria

Titles and abstracts of all search results were screened to include the studies that provided the clinical outcome-related information following the local or systemic supplementation of zoledronic acid in surgically managed GCTB. Only comparative studies with a control group were included for meta-analysis, and the remaining were considered for qualitative synthesis. Abstracts-only publications, animal studies, basic science/cellular studies, non-clinical studies, case reports, editorials, expert opinions, reviews, letters, and technical tips were excluded. Studies that did not analyze the tumor recurrence during the follow-up were also excluded.

Data Extraction

The primary author’s name, year of publication, level of evidence (as per JBJS [Journal of bone and joint surgery] guidelines), sample size, mode and dose of zoledronic acid administration, mean follow-up duration, and tumor recurrence rates were charted for each of the included studies. Additionally, the patients’ age, gender distribution, use of cement, extended curettage technique, primary/recurrent nature of the GCTB treated, and soft tissue extension were charted for the patients with recurrence and those that did not have a recurrence. The frequency of any major adverse events was also charted for the patients receiving zoledronic acid supplementation and those not receiving it. The discrepancies in data charting were settled through the reevaluation of the concerned studies and mutual discussion.

Quality and Risk of Bias Assessment

Two authors separately performed the risk of bias assessment. Conflicting opinions were settled through discussion and mutual consensus. The Newcastle-Ottawa Quality Assessment Scale was considered for evaluating non-randomized studies [12], and the Cochrane Risk of Bias Tool for Randomized Controlled Trials were considered for randomized studies [13].

Statistical Analysis

A meta-analysis of the included studies was performed using Review Manager (RevMan, Version 5.3, Copenhagen: The Nordic Cochrane Centre, Cochrane Collaboration, 2014). First, the dichotomous data based upon the data extraction criteria were compared between groups treated with and without supplementation of zoledronic acid using combined estimates of risk ratio (RR). Similarly, dichotomous data of the other aforestated parameters were compared between cases with recurrence and those without recurrence. Then, the continuous data between cases with recurrence and those without recurrence were analyzed using weighted mean differences (WMD) and 95% confidence intervals (95% CI). The fixed-effect model of analysis was used for comparisons with low heterogeneity (I^2^ < 50%), and the random-effect model of analysis was used for comparisons with high heterogeneity (I^2^ < 50%). A p-value of <0.05 was considered statistically significant.

Results

**Figure 1:**
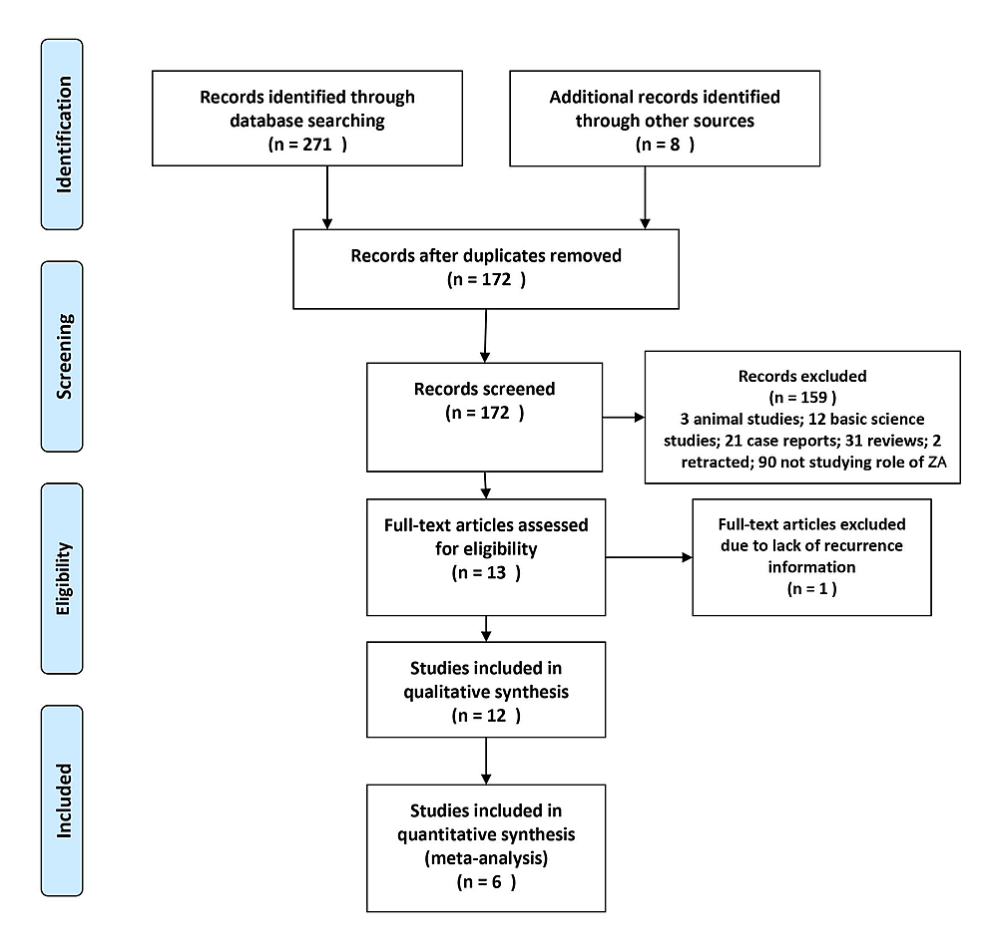
PRISMA (preferred reporting items for systematic reviews and meta-analyses) flow diagram for the current meta-analysis. ZA: Zoledronic acid.

The search resulted in 271 results (PubMed: 47, Embase: 135, Web of Science: 81, others: 8). After excluding duplicates (n = 99), the titles and abstracts of 172 papers were screened. Title and abstract screening resulted in 13 relevant studies that analyzed the clinical outcomes with zoledronic acid supplementation in surgically managed GCTB (Figure 1) [14-26]. However, six studies were excluded from the meta-analysis as those were case series without any control group (Table 1) [14-19], and one comparative study was excluded as it did not provide any tumor recurrence-related information [20]. As a result, six comparative studies were included in the current meta-analysis (Table 2). Five studies were non-randomized, and the risk of bias assessment using the Newcastle-Ottawa Quality Assessment Scale for the non-randomized studies suggested a good quality of the included studies, with all studies scoring ≥7 (Table 3). One study was an open-label phase II randomized control trial. Except for the obvious non-blinded nature of the trial, it had a low risk of bias in case selection, attrition bias, reporting, and other bias on Cochrane Risk of Bias Tool for Randomized Controlled Trials.

**Table 1:** Detailed characteristics of case series that studied the recurrence rates of GCTB with zoledronic acid supplementation and were excluded from the meta-analysis. ^# ^Minimum follow-up. ^~ ^10 patients underwent non-surgical management of recurrence/metastasis or primary tumor and the tumor size did not progress. GCTB: Giant cell tumor of bone.

S. No.	Authors	Year	Sample size	Zoledronic acid regimen	Mean follow-up	Recurrence rates
1.	Singaravadivelu et al. [17]	2020	10	Three doses of 4 mg zoledronic acid, one preoperative and two postoperative. Extended curettage was done three weeks after the preoperative dose of zoledronate	24 months^#^	0%
2.	Greenberg et al. [16]	2019	17	Zoledronic mixed bone cement: 4 mg/100 mL of zoledronic acid was added to each bag of bone cement	52 months	5.97%
3.	Gouin et al. [14]	2014	20	Five three-weekly injections of 4 mg zoledronic acid postoperatively	63 months	15%
4.	Nishisho et al. [18]	2015	5	Variable strength/doses, a variable waiting period before surgery (1-10 weeks)	19 months	20%
5.	Chen et al. [19]	2015	4	Zoledronic mixed bone cement: 4 mg of zoledronic acid + 1 gm vancomycin were added to each bag of bone cement	28 months	0%
6.	Balke et al. [15]	2010	17	Zoledronic acid (4 mg) was given intravenously as a single infusion in three cases and two infusions in three and six infusions in the remaining	23 months	17%^~^

**Table 2:** Comparative studies that investigated the recurrence rates of GCTB treated with zoledronic acid supplementation. * Cases undergoing extended curettage in the control group were considered. ^# ^Minimum follow-up. GCTB: Giant cell tumor of bone.

S. No.	Authors	Year	Level of evidence	Sample size	Zoledronic acid regimen	Mean follow-up	Recurrence rates
1.	Lipplaa et al. [21]	2019	I	Zoledronic acid group: 8	Postoperative intravenous zoledronic acid (4 mg) at 1, 2, 3, 6, 9, and 12 months after surgery.	93.5 months	Zoledronic acid group: 38%
				Control group: 6			Control group: 17%
2.	Kundu et al. [22]	2018	II	Zoledronic acid group: 18	Preoperative three doses of intravenous zoledronic acid (4 mg) at three-week intervals. The extended curettage was performed two weeks after the last infusion.	32 months	Zoledronic acid group: 5.5%
				Control group: 19			Control group: 21%
3.	Xu et al. [25]	2017	III	Zoledronic acid group: 7	One preoperative dose of intravenous zoledronic acid (4 mg) was given to each patient, and for a two-year period after surgery, patients received one dose at four-week intervals.	47.2 months	Zoledronic acid group: 28%
				Control group: 16			Control group: 43%
4.	Wei et al. [23]	2013	II	Zoledronic acid group: 28	One preoperative dose of intravenous zoledronic acid (4 mg) was given to each patient, one week before surgery, and one three weeks after surgery; after that 4 mg of zoledronic acid was given every four weeks for three years after surgery or till the patients could not tolerate it.	36 months^#^	Zoledronic acid group: NIL
				Control group: 25			Control group: 16%
5.	Gouin et al. [26]	2013	III	Zoledronic acid group:	Postoperative intravenous zoledronic acid (4 mg) every three weeks for three months was given after the surgical procedure.	72 months	Zoledronic acid group: 15.3%
				13 Control group: 39*			Control group: 30.7%
6.	Tse et al. [24]	2008	III	Zoledronic acid group: 24	Preoperative intravenous zoledronic acid (4 mg) two doses with each dose at an interval of three to four weeks between each dose; three more doses of zoledronic acid (4 mg) with each dose at an interval of three to four weeks and three months of additional oral clodronate.	48 months	Zoledronic acid group: 4.2%
				Control group: 20			Control group: 30%

**Table 3:** Quality assessment of the analyzed case-control studies according to Newcastle-Ottawa Quality Assessment Scale. * Valid representation of the scale parameter. - Unclear representation of the scale parameter.

S. No.	Authors	Is the case definition adequate?	Representativeness of the cases	Selection of controls	Definition of controls	Comparability	Ascertainment of exposure	Same method of ascertainment for cases and controls	Non-response rate
1.	Kundu et al. [2]	*	*	*	*	*	*	*	*
2.	Wei et al. [23]	*	*	*	*	*	*	*	*
3.	Xu et al. [25]	*	*	*	*	*	*	*	-
4.	Gouin et al. [26]	*	*	*	*	*	*	*	-
5.	Tse et al. [24]	*	*	*	*	*	*	*	-

Outcome Meta-Analysis

**Figure 2:**
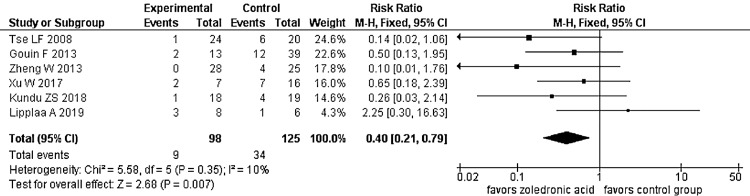
Forest plot of tumor recurrence in zoledronic acid supplemented group compared to the control group suggests significantly reduced recurrence in the former.

Influence of zoledronic supplementation on tumor recurrence: The detailed characteristics of the comparative studies included in this analysis are provided in Table 2. Pooled data of 223 cases suggested significantly lower recurrence rates in those receiving systemic or local zoledronic acid supplementation in the perioperative period (p = 0.007, details in Figure 2).

Association of other factors with tumor recurrence: Among the other factors that can potentially affect the recurrence rates, significant associations of higher recurrence rates were observed with non-usage of PMMA bone cement as a void filler (p = 0.002) and with the extraosseous or soft tissue extension of the tumor (p = 0.03) (Table 4). On the other hand, no significant associations with recurrence were observed with age, gender, and tumor presentation’s primary or recurrent nature.

**Table 4:** Factors influencing tumor recurrence in the reviewed studies. PMMA: Polymethyl methacrylate.

Variable	Included studies	Number of participants	Risk ratio (RR)/mean difference (MD)	95% Confidence interval	p-value	Remarks
Age	Gouin et al. [26]; Xu et al. [25]	228	MD = 2.56	[-0.59, 5.70]	0.11	No significant difference
Gender	Gouin et al. [26]; Xu et al. [25]	228	RR = 0.98	[0.69, 1.40]	0.93	No significant difference
Use of PMMA bone cement	Gouin et al. [26]; Tse et al. [24]	237	RR = 0.52	[0.35, 0.78]	0.002	Significantly lower recurrence with use of bone cement
Recurrent vs primary tumors	Gouin et al. [26]; Lipplaa et al. [21]	207	RR = 0.84	[0.46, 1.52]	0.56	No significant difference
Extraosseous/soft tissue extension	Lipplaa et al. [21]; Tse et al. [24]	58	RR = 4.42	[1.13, 17.24]	0.03	Significantly higher recurrence with extraosseous extension

No major adverse events/complications with zoledronic acid supplementation were reported in any of the reviewed studies. However, the majority of the studies reported minor fever and flu-like symptoms including fever, chills, bone pain, arthralgia, and myalgias with zoledronic acid administration.

Discussion

The current meta-analysis of comparative studies suggests an effective role of zoledronic acid supplementation in reducing recurrence rates in GCTB. The mode of zoledronic administration was through intravenous infusion with a 4 mg dose at a time. However, the number of repetition cycles varied among the studies, suggesting a lack of standardized protocol. Two studies used preoperative supplementation, two used postoperative supplementation, and three used both. Three studies were prospective, and three were retrospective. Besides zoledronic acid supplementation, the favorable thermogenic effect of cement in reducing recurrence and lower recurrence risk in contained GCTB lesions has been suggested by the statistically significant associations.

The currently available evidence concerning the use of zoledronic acid as an adjuvant in GCTB management suggests considerable progress in tumor recurrence reduction. Initially, the role of zoledronic acid in GCTB management was suggested by in-vitro studies that observed bisphosphonate-induced apoptosis of osteoclasts [27-29]. Cheng et al. [27], was the first to study the impact of bisphosphonate in in-vitro stromal GCTB cells. Zoledronate was the most potent reagent resulting in mean apoptosis of 27.41% stromal tumor cells. Several other authors later supported the findings in their in-vitro analysis of the impact of zoledronic acid on GCTB stromal cells [20,28,29]. However, the clinical evidence was still lacking until few authors reported case reports regarding the successful management of large GCTBs with zoledronic acid supplementation. Arpornchayanon et al. [30], in 2008, first reported a case of sacral GCTB managed with extended curettage following preoperative zoledronic supplementation. The results were favorable in terms of pain and with no recurrence at two years of follow-up. Subsequently, several other case reports and case series were published, which suggested a reduced incidence of recurrence with zoledronic acid supplementation [14-19]. Table 1 provides the case series details that used zoledronic acid supplementation but could not be included in our meta-analysis due to the lack of a control group.

Interestingly, both local (PMMA cement mixed) and systemic administration of zoledronic acid had reduced recurrence rates. Subsequently, most case-control studies also suggested reduced recurrence rates among GCTB patients undergoing zoledronic acid supplementation [22-26]. However, the efficacy of zoledronic acid in GCTB management has been questioned by the study by Lipplaa et al. [21], a small multicenter randomized phase II trial studying the role of adjuvant zoledronic acid versus placebo in advanced GCTB. The recurrence rate was 38% in the intervention group versus 17% in the control group, all occurring within 15 months postoperatively. Unfortunately, the study had its obvious limitations due to the very small sample size (n = 8 in zoledronic acid arm and n = 6 in placebo arm) and early closure of the trial due to the introduction of denosumab. In addition, there has been a lack of systematic reviews and meta-analyses on the role of zoledronic acid in GCTB. The current analysis fills that lacuna, comprehensively analyses the available evidence for the role of zoledronic acid supplementation in GCTB, and suggests a significant influence in reducing recurrence rates with a p-value as low as 0.007.

For the majority of the GCTB recurrences, a follow-up of 24-36 months is desirable to account for early as well as delayed presenting recurrences [31,32]. However, the mean follow-up duration in the reviewed studies ranged between 32 and 93.5 months. Thus, it can be assumed that within this follow-up, at least the majority of the recurrences would have been identified in the reviewed studies.

The tumor recurrences can be affected by several other parameters. Based on the previously studied factors and preliminary literature review, we analyzed the impact of age, gender, soft tissue extension, cement application, recurrent presentation, and curettage technique [33]. Our findings of the non-usage of PMMA bone cement as void filler and extraosseous/soft tissue extension resulting in higher recurrence rates have been supported by the previous studies [33,29]. However, the role of extended curettage in reducing tumor recurrence compared to the conventional techniques could not be analyzed in our analysis considering the recent nature of zoledronic acid supplementation when surgeons have already shifted to extended curettage techniques. Only in the study by Lipplaa et al. [21], four cases underwent traditional curettage without adjuvants. However, that was insufficient for the analysis as extended curettage was solely used in other studies.

Among the potent systemic therapies for GCTB, zoledronic acid (most potent among other bisphosphonates) and denosumab have been tried. Bisphosphonates affect vesicular trafficking, induce apoptosis in osteoclast-like giant cells in GCT, and cause apoptosis in neoplastic stromal cells [6]. Denosumab is a humanized monoclonal IgG2 antibody selectively targeting receptor activator of NF-κB (RANK) ligand (RANKL), which prevents RANKL from activating its receptor RANK on the surface of osteoclasts and their precursor, thus inhibits osteoclastic bone resorption [7]. Although the initial results were encouraging, later, several authors reported that the newly formed sclerotic bone and thickened cortex made curettage difficult. In addition, residual tumor cells may get trapped within the new bone, and the tumor may recur when denosumab is discontinued [9-11].

The studies included in the current meta-analysis suggested the effectiveness of systemic administration of the zoledronic acid in GCTB. However, there have been numerous reports of similar efficacy with local administration as well. The zoledronic acid can be mixed with PMMA cement and remains effective in tumor cells inhibition and apoptosis [19,29]. The only major concern regarding the administration of zoledronic acid is the lack of a standardized dosage regimen. The frequency and duration of zoledronic acid administration varied among the studies. Similarly, there is no standardized protocol regarding the amount of zoledronic acid mixed with PMMA cement. Probably, well-planned randomized controlled trials would be able to answer this.

Minor adverse events like fever, fatigue, and flu-like symptoms have been reported in very few cases with zoledronic acid supplementation [21]. No major complications have been reported with zoledronic acid supplementation in GCTB. The reason could be the short-term use in GCTB. Otherwise, long-term complications of bisphosphonates are well known and include musculoskeletal pain, atrial fibrillation, esophageal cancer, osteonecrosis of the jaw, and atypical femur fractures [34]. Lipplaa et al. [21] had reported one case of osteonecrosis of the jaw in a patient who had received systemic zoledronic acid supplementation. Still, the role of zoledronic acid in this complication could not be ascertained as the same patient had received 25 cycles of denosumab. Long-term studies would be required to rule out any delayed complications of short-term use of zoledronic acid.

The current analyses had some limitations. First, the number of studies included in the meta-analysis synthesis was small. The number could have been increased by including case series, but that would have affected the quality of inferences, and thus only comparative studies were included. Therefore, the case series were included in the qualitative review. Second, the surgical techniques and surgeons’ expertise in tumor handling can affect tumor outcomes. Also, the type of adjuvants used in extended curettage varied. The impact of these technical aspects could not be analyzed due to limited information of center-based and surgeon-based variations. The inclusion of comparative studies reduces this bias to some extent. Third, the zoledronic acid dosage and duration varied among the studies. Thus, an effective regimen cannot be predicted from the available information. Fourth, we analyzed the major other contributing factors in tumor recurrence. Still, additional potential patients or treatment-related factors could have contributed to the recurrence. Fifth, the tumor location-specific recurrence rates could not be analyzed in this study. Lastly, there is a lack of well-planned randomized controlled trials that could provide top-quality evidence. Nevertheless, the current analysis predicts the effectiveness of zoledronic acid at a very low p-value, which is highly unlikely to be because of chance. Additionally, the analysis fulfills the need for a scientific discussion concerning zoledronic acid usage in GCTB in the current scenario of lack of consensus.

## Conclusions

To conclude, the current systematic review suggests that zoledronic acid supplementation improves tumor recurrence rates in surgically treated GCTB. Therefore, we recommend using zoledronic acid in systemic or local form following aggressive extended curettage of the tumor. However, considering the limited and low-level evidence available, well-planned randomized controlled trials are needed to predict an effective regimen for its supplementation and sound recommendations.
